# Changes in Soil Microbial Communities across an Urbanization Gradient: A Local-Scale Temporal Study in the Arid Southwestern USA

**DOI:** 10.3390/microorganisms9071470

**Published:** 2021-07-09

**Authors:** Yongjian Chen, Adalee Martinez, Sydney Cleavenger, Julia Rudolph, Albert Barberán

**Affiliations:** Department of Environmental Science, University of Arizona, Tucson, AZ 85721, USA; adaleemartinez@email.arizona.edu (A.M.); sydneycleavenger@email.arizona.edu (S.C.); juliarudolph@email.arizona.edu (J.R.); barberan@email.arizona.edu (A.B.)

**Keywords:** urbanization, temporal dynamics, arid ecosystems, bacteria/archaea, fungi

## Abstract

Urban development is one of the leading causes of biodiversity change. Understanding how soil microorganisms respond to urbanization is particularly important because they are crucial for the provisioning of ecosystem functions and services. Here, we collected monthly soil samples over one year across three locations representing an urbanization gradient (low-moderate-high) in the arid Southwestern USA, and we characterized their microbial communities using marker gene sequencing. Our results showed that microbial richness and community composition exhibited nonsignificant changes over time regardless of the location. Soil fungal richness was lower in moderately and highly urbanized locations, but soil bacterial/archaeal richness was not significantly different among locations. Both bacteria/archaea and fungi exhibited significant differences in community composition across locations. After inferring potential functional groups, soils in the highly urbanized location had lower proportions of arbuscular mycorrhizal fungi and soil saprotrophic fungi but had higher proportions of bacterial taxa involved in aromatic compound degradation, human pathogens, and intracellular parasites. Furthermore, ammonia-oxidizing bacteria were more abundant in the highly urbanized location, but ammonia-oxidizing archaea were more abundant in lowly and moderately urbanized locations. Together, these results highlight the significant changes in belowground microbial communities across an urbanization gradient, and these changes might have important implications for aboveground–belowground interactions, nutrient cycling, and human health.

## 1. Introduction

Urban development is modifying natural landscapes, as well as altering biodiversity and ecosystem services, at an unprecedented rate [1]. Currently, 55% of the global population is living in urban areas, and this proportion is projected to increase to 68% by 2050 [2]. Residents of urban landscapes live at high densities and built structures cover most of the land surface, resulting in significant modifications to the natural environment [3]. For example, in arid ecosystems, green-space fragmentation can impact biodiversity through natural habitat reduction and net primary production changes [4]. One major ecological consequence of urbanization is biotic homogenization, in which ecological communities become progressively more similar due to human-induced landscape simplification or species invasions [5]. Most research has focused primarily on understanding how plants and animals either adapt or go extinct due to urban pressures such as increased nutrient inputs, atmospheric pollution, and habitat degradation [6]. However, the consequences of urbanization on microbial communities have received little attention (but see [7]).

Studying how microorganisms respond to urbanization is particularly relevant given their fundamental roles in maintaining ecosystem functions and services [8], notably human health and well-being [9]. On the one hand, some microbes can cause infectious diseases to humans by acting as important pathogens [10]. On the other hand, it has been proposed that the positive health effects of proximity to nature might be attributable to the exposure to microbial diversity that builds up the antigenic repertoire of the immune system [11].

Urbanization can have significant impacts on microbial richness and community composition. Previous studies have shown that urbanization often leads to decreasing geographic variability in microbial community composition [12,13]. However, the effects of urbanization can vary among microbial taxonomic groups. For example, soil fungal richness was found to decrease in highly urbanized areas, but bacterial richness showed no response [13]. Another soil study showed that fungal richness and community composition did not differ with urban stress, whereas bacterial community composition did [14]. Furthermore, urban development in arid ecosystems can alter soil microbial carbon and nutrient cycling by shaping the types of microorganisms found in urban habitats [15]. While these studies have provided significant insights into the ecological impacts of urbanization on microbial communities, it remains largely unknown whether or not the temporal dynamics of microbial communities change across urbanization gradients. It has been argued that the richness and community composition of microorganisms can vary across days, seasons, and years [16,17,18,19]. Such temporal changes in microbial communities can reflect community stability and have important implications for predicting microbial responses to disturbances [20]. Therefore, a better understanding of the ecological impacts of urbanization on microorganisms requires longitudinal studies that examine how the temporal dynamics of microbial communities vary across urbanization gradients.

**Figure 1:**
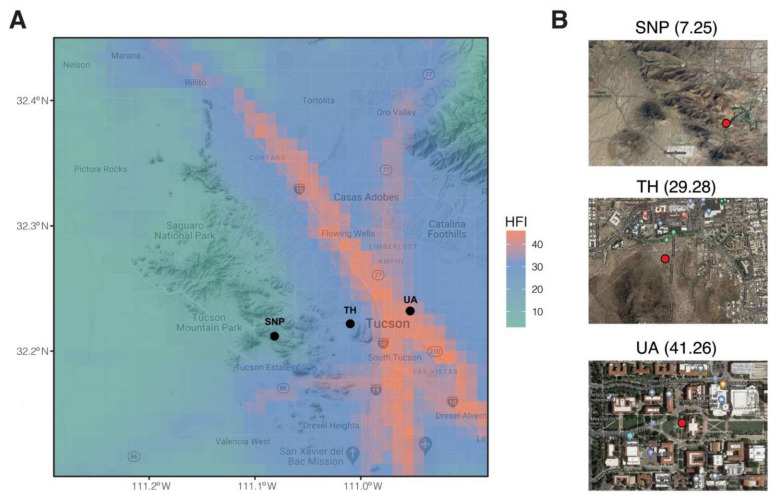
Sampling locations in this study. (**A**) Map is overlayed by human footprint index (HFI). Black points denote sampling locations. (**B**) A zoomed view of each sampling location. Red points denote sampling plots. Numbers in brackets are human footprint index. SNP: Saguaro National Park. TH: Tumamoc Hill. UA: University of Arizona.

Globally, arid ecosystems constitute 41% of the land surface and are home to over 38% of the human population, and these numbers are likely to increase substantially as a consequence of desertification and the projected population growth [21]. The arid Southwestern USA is experiencing one of the fastest population growths in the country [22]. Denser populations in arid areas with higher urbanization pressure require solutions that consider the role of environmental microorganisms. Here, we collected monthly soil samples over one year across three locations representing an urbanization gradient in Tucson, Arizona, USA (Figure 1). We characterized the soil bacterial/archaeal and fungal communities by sequencing environmental DNA in order to examine if the soil microbial richness and community composition, as well as their temporal dynamics, change across the urbanization gradient.

## 2. Materials and Methods

### 2.1. Study Locations

We selected three locations along an urbanization gradient in Tucson, Arizona, USA (Figure 1). The University of Arizona’s garden by the Old Main Building (32.232° N, 110.953° W) was chosen as our most urbanized sampling location. Old Main’s garden is composed of plants chosen based on the Sonoran Desert landscape, including saguaro (*Carnegiea gigantea*), ocotillo (*Fouquieria splendens*), soaptree yucca (*Yucca elata*), Parry’s agave (*Agave parryi*), and pink fairy duster (*Calliandra eriophylla*) (https://apps.cals.arizona.edu/arboretum/map/; accessed on 3 October 2018). The University of Arizona campus is near downtown Tucson and is highly trafficked by its ~45,000 enrolled students. Tumamoc Hill (32.226° N, 111° W) is a nature preserve and field station located west of downtown Tucson (https://tumamoc.arizona.edu/; accessed on 3 October 2018) with a typical Sonoran Desert vegetation dominated by saguaro, mesquite (genus *Prosopis*), blue palo verde (*Parkinsonia florida*), creosote bush (*Larrea tridentata*), and barrel cactus (*Ferocactus wislizeni)* [23]. Our least urbanized sampling location was Saguaro National Park (32.279° N, 111.145° W). This national park (https://www.nps.gov/sagu/; accessed on 3 October 2018) supports high biodiversity and is dominated by saguaros, mesquite shrubs, palo verde, and creosote bush [24]. The urban intensity of the three locations was assessed by the human footprint index at a resolution of 30 arc-seconds (i.e., 1 km × 1 km). This index incorporates information on population density, built-up environments, electric power infrastructure, pasture lands, crop lands, roads, railways, and navigable waterways to estimate the footprint of human influence [25]. The human footprint index of the University of Arizona, Tumamoc Hill, and Saguaro National Park is 41.26, 29.28, and 7.25, respectively (Figure 1).

### 2.2. Soil Sampling

We established a 10 × 10 m plot within each of the three sampling locations. Within the 10 × 10 m plot, three soil samples (0–10 cm depth) were taken at random positions on a monthly basis from October 2018 to October 2019 (June 2019 was excluded). This resulted in a total of 108 soil samples (3 samples × 1 plot × 3 locations × 12 months). Soil samples were placed in sterile Whirl-Pack bags and packed on ice, and they were transported to the University of Arizona laboratory immediately after collection. Soil samples were sieved through a 2 mm mesh to remove roots and large debris and were then homogenized. All soil samples were stored in a −80 °C freezer until molecular analyses were performed.

### 2.3. Molecular Analyses

Total soil genomic DNA was extracted using a DNeasy PowerLyzer PowerSoil Kit (Qiagen, Hilden, Germany) according to the manufacturers’ instructions. To characterize the bacterial and archaeal communities, the V4 hypervariable region of the 16S rRNA gene was amplified via PCR using the 515-F (GTGCCAGCMGCCGCGGTAA) and 806-R (GGACTACHVGGGTWTCTAAT) primer pair [26]. To characterize the fungal communities, the first internal transcribed spacer (ITS1) region of the rRNA operon was amplified by PCR using the ITS1-F (CTTGGTCATTTAGAGGAAGTAA) and ITS2 (GCTGC GTTCTTCATCGATGC) primer pair [27]. The primers included the Illumina adapters with the reverse primers also having an error-correcting 12-bp barcode unique to each sample to permit demultiplexing. Negative controls without the DNA template were included in each batch of PCR reactions to check for possible contamination. Each PCR was performed in duplicate. PCR products were cleaned using an UltraClean PCR Clean-Up Kit (MoBio Laboratories, Carlsbad, CA, USA) and quantified fluorescently with the Quant-iT PicoGreen dsDNA Assay Kit (Invitrogen, Carlsbad, CA, USA). The purified PCR products from all samples were pooled together in equimolar concentrations and sequenced on a 2 × 150 bp Illumina MiSeq platform (Illumina, San Diego, CA, USA). All sequencing runs were conducted at the Microbiome Core, Steele Children Research Center, University of Arizona.

### 2.4. Sequence Processing

Raw reads were demultiplexed using idemp (https://github.com/yhwu/idemp; accessed on 16 February 2019). Then, raw reads were processed using DADA2 [28], which can resolve exact biological sequences by assembling reads into error-corrected amplicon sequence variants (phylotypes hereafter). The DADA2 pipeline included quality filtering, modeling of error rate, dereplication, phylotype inference, merging of paired-end reads, construction of phylotype count table, chimera removal, and taxonomy assignment. There were three differences between the DADA2 pipelines for 16S and ITS reads. First, for the ITS reads, cutadapt [29] was used to remove primer sequences prior to quality filtering because of the length variation in the ITS region. Second, during quality filtering, the 16S reads, but not the ITS reads, were truncated to the same length. This is because some ITS variants might be shorter than the truncation length. Third, the taxonomic identities of 16S and ITS phylotypes were determined using the RDP classifier [30] with a confidence threshold of 0.5 trained on the SILVA nr version 132 database [31] and the UNITE database [32], respectively. The phylotypes present in negative controls were removed. Those 16S phylotypes without a bacterial or archaeal domain assignment and assigned to chloroplast or mitochondrial origin were removed. Those ITS phylotypes without a fungal domain assignment were removed. To remove the shallowly sequenced samples, we discarded samples with less than 10,000 sequences, leaving a total of 101 samples for bacteria/archaea and 104 samples for fungi. The sequence counts were normalized using a cumulative-sum scaling [33]. The putative functions of bacterial/archaeal phylotypes were inferred using functional annotation of prokaryotic taxa (FAPROTAX; [34]). The putative fungal functional guilds were inferred using FUNGuild [35]. We retained those fungal phylotypes assigned to a single guild and that had a “highly probable” or “probable” confidence ranking.

### 2.5. Statistical Analyses

Statistical analyses were implemented in R (version 4.0.2) [36] using the vegan [37], agricolae [38], and FSA [39] packages. Both bacterial/archaeal and fungal phylotype richness were normally distributed according to the Shapiro–Wilk test (bacteria/archaea: *p* = 0.73; fungi: *p* = 0.12), and the homogeneity of variances was confirmed using the Levene’s test (bacteria/archaea: *p* = 0.95; fungi: *p* = 0.61). Therefore, we conducted a two-way analysis of variance (ANOVA) followed by a Tukey’s honestly significant difference (HSD) post hoc test to examine if phylotype richness was significantly different among locations and time. To test if community dissimilarity (Bray–Curtis metric) was significantly different among locations and time, we conducted a two-way permutational multivariate analysis of variance (PERMANOVA). A Mantel test with the Pearson method was performed to explore the association between community dissimilarity and temporal distance. The proportions of taxonomic and functional groups (based on proportional numbers of 16S or ITS sequences) did not meet the assumptions of normality and homogeneity of variances (*p* < 0.05 in both Shapiro–Wilk and Levene’s tests). Therefore, we conducted a nonparametric Kruskal–Wallis test followed by a Dunn’s post hoc test to examine if the proportions of taxonomic and functional groups were significantly different among locations. *p*-values for multiple testing were corrected using the Benjamini–Hochberg method [40].

## 3. Results

### 3.1. General Description of Microbial Communities

After quality filtering, we obtained 8,648,018 sequences for soil bacteria/archaea and 5,858,931 sequences for soil fungi. These sequences were assembled into a total of 31,887 bacterial/archaeal phylotypes (Table S1) and 6832 fungal phylotypes (Table S2). On average, 1558 bacterial/archaeal phylotypes and 240 fungal phylotypes were detected per sample, with the range being 438–2443 for bacteria/archaea and 86–503 for fungi. At the class level, soil bacterial communities were dominated by *Alphaproteobacteria* (range: 9.0–38.5%), *Actinobacteria* (range: 6.4–25.8%), *Thermoleophilia* (range: 3.0–21.7%), *Gammaproteobacteria* (range: 1.9–8.5%), and *Rubrobacteria* (range: 0.7–15.4%) across all samples (Table S3). For fungal communities, the classes *Dothideomycetes* (range: 9.2–76.6%), *Sordariomycetes* (range: 7.3–67.6%), *Agaricomycetes* (range: 0.06–49.6%), and *Eurotiomycetes* (range: 0.06–14.7%) were the dominant lineages across all samples (Table S4).

### 3.2. Effects of Location and Time on Predicting Microbial Richness and Community Composition

**Figure 2:**
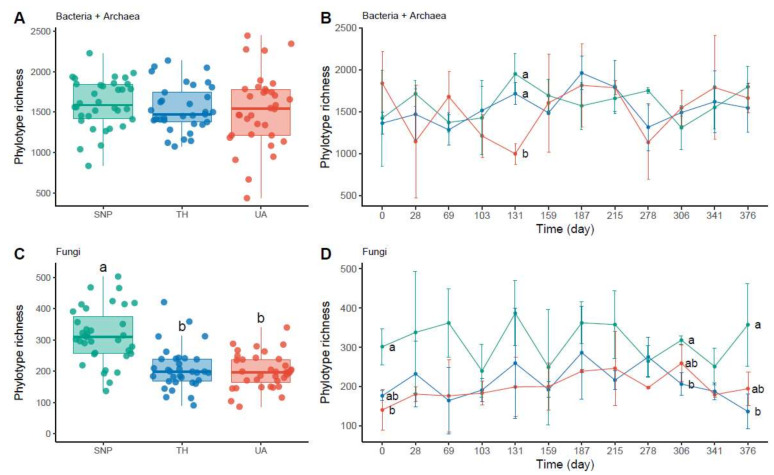
Changes in microbial richness across locations. Different letters indicate significant difference (*p* < 0.05) based on Tukey’s HSD test. (**A**,**C**) Phylotype richness difference across locations. (**B**,**D**) Temporal change in phylotype richness. Horizontal axis denotes sampling day. Points are means of phylotype richness per day and error bars are standard deviations of the mean. Location abbreviations are the same as those in Figure 1.

Soil bacterial/archaeal phylotype richness was not significantly different among locations or time (two-way ANOVA: location: *F*_2,95_ = 0.43, *p* = 0.65, time: *F*_1,95_ = 1.44, *p* = 0.23, location × time: *F*_2,95_ = 0.08, *p* = 0.93; Figure 2A,B). Only at the 131st day of sampling, soil bacterial/archaeal communities were less diverse in the University of Arizona than in Tumamoc Hill and Saguaro National Park (Figure 2B). In contrast, soil fungal phylotype richness was significantly different among locations but not time (two-way ANOVA: location: *F*_2,98_ = 27.38, *p* = 3.6 × 10^−10^; time: *F*_1,98_ = 0.11, *p* = 0.74; location × time: *F*_2,98_ = 0.96, *p* = 0.39; Figure 2C,D). Soil fungal richness was lower in the University of Arizona and Tumamoc Hill than in Saguaro National Park (Figure 2C). This pattern was also observed at the beginning and near the end of the sampling period (the 0th, 306th, and 376th days; Figure 2D).

**Figure 3:**
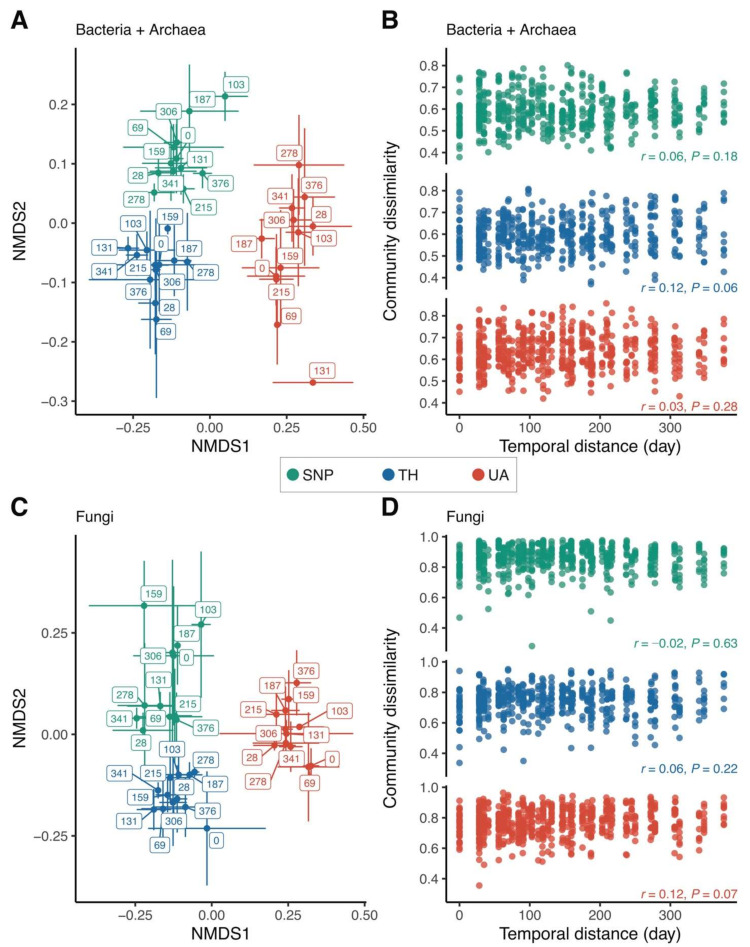
Changes in microbial community composition across locations. (**A**,**C**) Nonmetric multidimensional scaling (NMDS) ordinations show that samples are well-clustered by location. Label numbers denote sampling days. Points are means of NMDS axes per day and error bars are standard deviations of the mean. (**B**,**D**) Relationship between community dissimilarity and temporal distance. Mantel test (Pearson method) statistics are shown. Location abbreviations are the same as those in Figure 1.

Location explained a much larger variation in bacterial/archaeal community composition than time (two-way PERMANOVA: location: *F*_2,95_ = 19.40, *p* = 0.001, *R*^2^ = 0.28, time: *F*_1,95_ = 2.28, *p* = 0.008, *R*^2^ = 0.02, location × time: *F*_2,95_ = 1.50, *p* = 0.04, *R*^2^ = 0.02; Figure 3A). There was no directional change in bacterial/archaeal community composition over time; samples collected closer in time did not have similar bacterial/archaeal community compositions (Figure 3A), and there was no significant correlation between bacterial/archaeal community dissimilarity and temporal distance (Mantel test: *p* > 0.05 in all locations; Figure 3B). Likewise, location was more important than time in predicting fungal community composition (two-way PERMANOVA: location: *F*_2,98_ = 11.40, *p* = 0.001, *R*^2^ = 0.18, time: *F*_1,98_ = 1.22, *p* = 0.12, *R*^2^ = 0.01, location × time: *F*_2,98_ = 1.28, *p* = 0.07, *R*^2^ = 0.02; Figure 3C). Soil fungal community composition did not change in any particular direction over time (Mantel test: *p* > 0.05 in all locations; Figure 3D). The significant effect of location on predicting microbial community composition was also mirrored by the proportional changes of the dominant microbial lineages across the three locations. Compared to Tumamoc Hill and Saguaro National Park, soil bacterial communities from the University of Arizona exhibited higher proportions of *Alphaproteobacteria* and *Gammaproteobacteria*, but lower proportions of *Thermoleophilia* and *Rubrobacteria* (Figure S1A). For fungi, soils from the University of Arizona had lower proportions of *Dothideomycetes*, *Eurotiomycetes*, and *Pezizomycetes*, but a higher proportion of *Tremellomycetes* (Figure S1B).

### 3.3. Proportional Changes in Microbial Functional Groups across Locations

**Figure 4:**
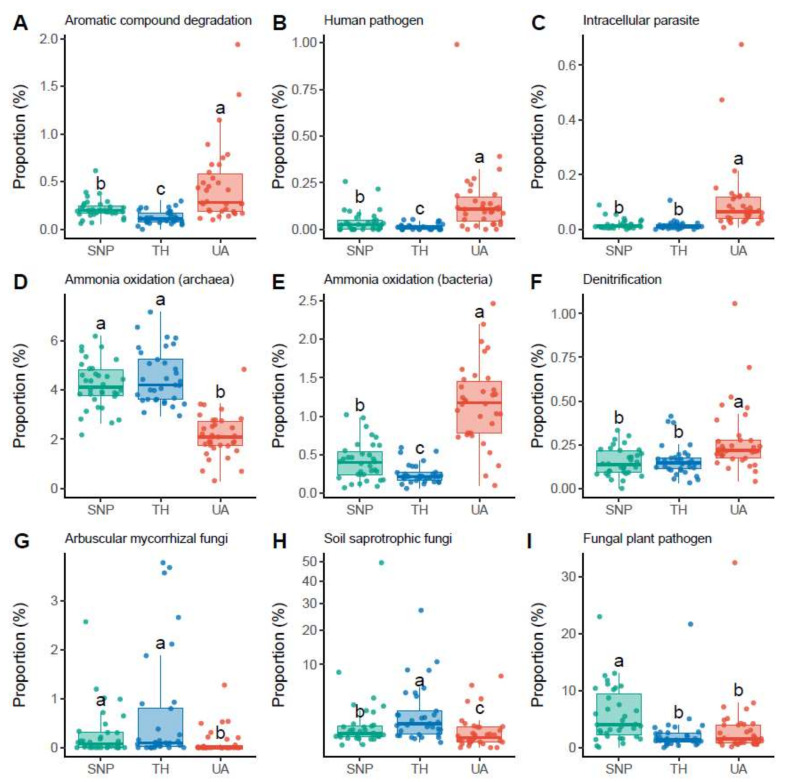
Changes in the proportions of microbial functional groups across locations. Different letters indicate significant differences (*p* < 0.05) based on Dunn’s test. (**A**–**F**) Dominant bacterial/archaeal functional groups. (**G**–**I**) Dominant fungal functional groups. Results of other functional groups are provided in Supporting information (Figures S2 and S3). Location abbreviations are the same as those in Figure 1.

The proportions of soil microbial functional groups also exhibited significant differences across the three locations (Figure 4, Figures S2 and S3). For the inferred functional groups of bacteria/archaea, soils from the University of Arizona had a higher proportion of bacteria associated with aromatic compound degradation (mainly *Nocardioides* sp.; Figure 4A), human pathogens (mainly *Roseomonas* sp. and *Coxiella* sp.; Figure 4B), and intracellular parasites (mainly *Rickettsiales* sp. and *Legionella* sp.; Figure 4C). In addition, archaea associated with ammonia oxidation (mainly the family *Nitrososphaeraceae*) were more abundant in Saguaro National Park and Tumamoc Hill (Figure 4D), whereas bacteria associated with ammonia oxidation (mainly the family *Nitrosomonadaceae*) were more abundant in the University of Arizona (Figure 4E). Soils from the University of Arizona also had a higher proportion of bacteria associated with denitrification (mainly *Rhodoplanes* sp.; Figure 4F). For the inferred functional groups of fungi, soils from the University of Arizona had lower proportions of arbuscular mycorrhizal fungi (mainly *Rhizophagus* sp. and *Funneliformis* sp.) and soil saprotrophic fungi (mainly *Tulostoma* sp.) than those from Tumamoc Hill and Saguaro National Park did (Figure 4G,H). In addition, the proportion of fungal plant pathogens (mainly *Thanatephorus* sp., *Monosporascus* sp., and *Curvula* sp.) was significantly lower in soils from the University of Arizona than in those from Saguaro National Park (Figure 4I).

## 4. Discussion

Present and historic land use can alter the microbial diversity, composition, and functioning of soil ecosystems [41,42,43]. In drylands, land-use changes such as urbanization can promote irreversible regime shifts related to soil degradation [44]. Dryland soil degradation is a serious environmental challenge associated with decreased aboveground and belowground biodiversity, plant productivity, loss of carbon storage in soil, and dust-related health hazards such as allergies and automobile accidents caused by dust storms [45,46]. Studying the changes in microbial communities across urbanization gradients can provide significant insights into the impacts of land-use changes on ecosystem functions and services, as well as the health of humans, animals, and plants. Here, we studied the temporal richness and composition patterns of soil microbial communities across three locations with different urbanization pressures in the arid Southwestern USA.

### 4.1. Nonsignificant Temporal Patterns of Microbial Richness and Community Composition

Compared to previous studies only focusing on examining the impact of urbanization on the spatial variability of microbial communities [47,48,49,50], we sampled monthly in triplicates to test if temporal dynamics of soil microbial communities change across the urbanization gradient. The temporal dynamics of ecological communities are intrinsically complex because community assembly processes such as environment filtering, species interactions, dispersal, and ecological drift change temporally [51]. These ecological processes modulate the temporal dynamics of species colonization and exclusion [52], ultimately leading to temporal changes in species richness and community composition. While several studies have identified predictable changes in microbial communities over time [16,17,18], other studies have shown that microbial communities exhibited nonsignificant or weak temporal patterns [20,53]. For instance, a meta-analysis showed that microbial community composition did not exhibit significant temporal patterns in more than half of the time-series studies examined [20]. In our study, neither soil bacterial/archaeal richness nor fungal richness exhibited predictable changes over time. Although time was a significant predictor of bacterial/archaeal community composition, it only explained a negligible variation. In addition, we found that community dissimilarities of bacteria/archaea and fungi were not related to temporal distance, which is in contrast to previous studies showing that community dissimilarities tend to decrease over time (i.e., time-decay relationship; [54,55]).

The lack of significant temporal patterns is surprising given the monsoon rains experienced in the Southwestern USA. From July to mid-September, the summer monsoon brings a pronounced increase in rainfall that can be up to 50% of the total annual precipitation [56]. This monsoon can lead to a seasonal change in vegetation [57] and is the primary dust-raising meteorological event [58]. Although dryland soil microbial communities tend to be temporally stable both in richness and composition and are more variable spatially [59], we would expect that a dramatic event such as summer monsoon precipitation would trigger a large and rapid response at the community level [60]. However, previous studies have reported mixed results, from minor microbial community changes [61] to large effects of monsoon precipitation [62].

Temporal patterns of microbial communities can be sensitive to the study duration and sampling intensity. It has been suggested that microbial communities exhibit the most dramatic changes within ranges of one day to one month [20]. Therefore, a possible reason for the nonsignificant temporal patterns is that our monthly sampling did not capture the within-month variation of microbial communities. Perhaps more importantly, the observed temporal patterns in this study might be dampened by the presence of relic DNA. Relic DNA is the DNA derived from dead microbial cells or secreted extracellular DNA and it is well known to account for a large fraction of microbial DNA in soils [63]. As relic DNA represents those metabolically inactive microorganisms that can persist in the environment over a long time, the presence of relic DNA in our soil samples might attenuate the true temporal patterns. For instance, a recent study showed that removing relic DNA using a propidium monoazide treatment can enhance the detection of significant temporal patterns of soil microbial communities [64]. Future studies increasing sampling intensity and removing relic DNA are required to better understand the changes in temporal dynamics of soil microbial communities across urbanization gradients.

### 4.2. Changes in Microbial Richness and Community Composition across the Urbanization Gradient

While we did not observe any significant temporal patterns, our study showed some interesting results regarding the changes in microbial richness and community composition across the urbanization gradient. Soil fungal richness was significantly lower in the University of Arizona (highly urbanized site) and Tumamoc Hill (moderately urbanized site) than in Saguaro National Park (lowly urbanized site). This is consistent with a global study showing that soil fungal communities are less diverse in more urbanized areas [13]. The decreasing soil fungal richness across the urbanization gradient can be driven by many factors such as changes in soil properties, but one of the most important factors is the reduced plant species richness. In urban habitats, plant species are selected exclusively to meet a relatively narrow set of human needs [5]. In addition, urban areas have a fragmented mosaic landscape pattern characterized by small and disconnected green spaces, resulting in the absence of certain plant species [65]. Such reduced plant species richness might limit fungal richness because fungi are prone to establish close associations with plants [66,67]. In contrast, our results showed that soil bacterial/archaeal richness was not significantly different among the three locations. This is consistent with previous studies showing that urbanization has no impact on the richness of bacteria/archaea [12,14,47]. Taken together, these results suggest that soil fungal richness may be more susceptible to urbanization pressures than bacterial/archaeal richness.

Both soil bacterial/archaeal and fungal community composition exhibited significant differences across the urbanization gradient. These results are particularly relevant for bacteria/archaea because their richness was not impacted by urbanization, but their community composition was impacted. This means that the type and/or relative abundance of bacterial/archaeal taxa could be undergoing significant changes despite no net change in the number of bacterial/archaeal taxa [68]. Similar patterns were observed when studying the impact of urbanization on the woody plant communities in a neotropical savanna, where plant community composition, but not species richness, was significantly altered by urbanization [69]. Likewise, a previous study conducted in Manhattan showed that highly and lowly urbanized habitats had distinct soil bacterial community composition, yet had similar bacterial richness [14]. In contrast, the significant impacts of urbanization on both richness and community composition were documented in atmospheric and soil fungal communities [70]. Thus, richness and community composition are complementary indicators of the rate and pattern of the microbial response to urbanization.

### 4.3. Changes in the Proportions of Microbial Functional Groups across the Urbanization Gradient

The significant changes in microbial community composition across the urbanization gradient were associated with significant changes in the proportions of several important microbial functional groups. The proportions of plant-associated fungi, including arbuscular mycorrhizal fungi, fungal plant pathogens, and saprotrophic fungi, were significantly lower in soils in the University of Arizona compared to those in Saguaro National Park. For instance, both *Rhizophagus* sp. and *Funneliformis* sp. (arbuscular mycorrhizal fungi) can form obligate symbioses that are crucial for plant establishment [71]. In natural areas, root exudates in the top layer of soil can release chemical signals and substrates that recruit plant-associated fungi [8]. However, this effect might be weaker in urban areas because of the lower plant diversity [67].

Soil bacterial communities from the University of Arizona had a higher proportion of taxa associated with aromatic compound degradation. For example, *Nocardioides* sp. has been found to play key roles in degrading anthropogenic sources of polycyclic aromatic hydrocarbons [72]. In addition, soils from the University of Arizona had a higher proportion of bacterial taxa acting as potential human pathogens and intracellular parasites. To illustrate, *Coxiella* sp. can serve as triggers of query fever in humans [73]. We want to acknowledge that the presence of a potential pathogen sequence does not unequivocally indicate the presence in an active state of a disease-causing microorganism. Notwithstanding and consistent with our findings, urban soils in New York City’s Central Park were found to have a higher relative abundance of human pathogens than in nonurban soils [74]. A plausible reason is that in urban areas, there are more pathogens/commensals shed by humans via skin and respiratory droplets.

Our results suggest that the soil bacterial/archaeal taxa associated with nitrogen cycling might change across the urbanization gradient. Nitrogen dynamics are unique in drylands. In contrast to mesic ecosystems where nitrogen required by plants is supplied through soil organic matter (SOM) decomposition, plant growth and SOM decomposition are decoupled in drylands [75]. One of the most essential steps in nitrogen cycling is nitrification, where ammonia is first oxidized to nitrite and then nitrite is oxidized to nitrate. Ammonia oxidation is a rate-limiting step and is catalyzed by those bacteria and archaea having genes encoding ammonia monooxygenase. Our results showed that ammonia-oxidizing bacteria were more abundant in soils from the University of Arizona, whereas ammonia-oxidizing archaea were more abundant in soils from Tumamoc Hill and Saguaro National Park. Previous experimental and observational studies have shown that ammonia-oxidizing archaea were more likely to predominate in oligotrophic environments (e.g., low ammonium content), but ammonia-oxidizing bacteria tended to dominate in copiotrophic environments (e.g., high ammonium content) [76,77]. Therefore, it is possible that soils from the University of Arizona are watered and fertilized regularly, and thus, favor ammonia-oxidizing bacteria. Moreover, another essential step in nitrogen cycling is denitrification, which is a facultative respiratory pathway where nitrate is reduced to gaseous nitrogen species including dinitrogen, nitric oxide, and nitrous oxide. In particular, nitrous oxide is a potent greenhouse gas with significant contribution to global warming [78]. Previous studies have also shown that the urban heat island effect and urban management practices (e.g., fertilization and irrigation) can increase the greenhouse gas emission by accelerating the rates of denitrification in urban soils [79,80,81]. It is worth nothing, however, that the types of bacterial/archaeal taxa associated with nitrification and denitrification were inferred by their taxonomy, and thus, their abundances might be underestimated by our taxonomic marker gene sequencing approach. Future studies quantifying the abundance of nitrogen-related genes are required to provide a more detailed and comprehensive understanding of the effect of urbanization on soil microbial nitrogen cycling.

## 5. Conclusions

By characterizing the temporal dynamics of belowground microbial communities in three locations representing an urbanization gradient, we found that microbial communities did not exhibit significant changes over time but changed significantly across locations. These changes in belowground microbial communities might have important implications for critical ecosystem functions and services. Our study was conducted at a local scale in the arid Southwestern USA, and thus, the generalizability of our results needs to be further examined using data collected at larger scales and in other habitats. Urbanization necessitates the development of a research agenda on soil microbiomes to inform policy and management.

## Data Availability

The raw sequencing data have been deposited in the NCBI Sequence Read Archive under BioProject accession code PRJNA729590.

## Supplementary Materials

The following are available online at https://www.mdpi.com/article/10.3390/microorganisms9071470/s1, Figure S1: Changes in the proportions of taxonomic groups (class level) across locations, Figure S2: Changes in the proportions of bacterial/archaeal functional groups across locations, Figure S3: Changes in the proportions of fungal functional groups across locations, Table S1: Taxonomic identities of bacterial/archaeal phylotypes, Table S2: Taxonomic identities of fungal phylotypes, Table S3: A summary of the proportions of bacterial/archaeal classes, Table S4: A summary of the proportions of fungal classes.
