# Supplementary material for: Changes in Soil Microbial Communities across an Urbanization Gradient: A Local-Scale Temporal Study in the Arid Southwestern USA
Source: Microorganisms. 2021 Jul 9;9(7):1470. doi: 10.3390/microorganisms9071470 (PMC8305102; doi:10.3390/microorganisms9071470)
Supplement: Supplementary file 1 [file microorganisms-09-01470-s001.zip › SI.pdf]

**Supporting information for**

# Changes in soil microbial communities across an urbanization gradient: A local-scale temporal study in the arid Southwestern USA

Yongjian Chen[1*], Adalee Martinez[1], Sydney Cleavenger[1], Julia Rudolph[1], Albert Barberán[1]

[1]Department of Environmental Science, University of Arizona, Tucson, AZ 85721, USA

[*]**Corresponding author:**

Yongjian Chen (chenyj@email.arizona.edu)

**This PDF file includes:**

Table S1 to S4

Figure S1 to S3

**Table S1.** Taxonomic identities of bacterial/archaeal phylotypes.

**Table S2.** Taxonomic identities of fungal phylotypes.

**Table S3.** A summary of the proportions of bacterial/archaeal classes.

**Table S4.** A summary of the proportions of fungal classes.

Table S1, Table S2, Table S3, and Table S4 are available online as separate excel files under the Supporting Information for this article.

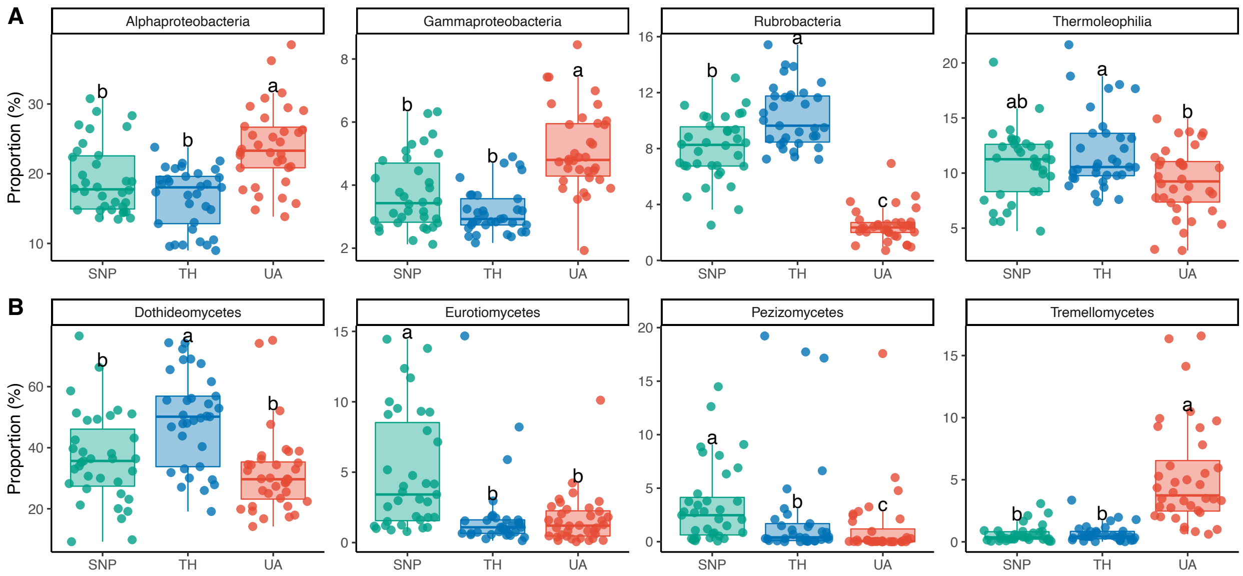


**Figure S1. Changes in the proportions of taxonomic groups (class level) across locations.** Different letters indicate significant differences ($P < 0.05$) based on Dunn's test. (A) Dominant bacterial classes. (B) Dominant fungal classes. SNP: Saguaro National Park. TH: Tumamoc Hill. UA: University of Arizona.

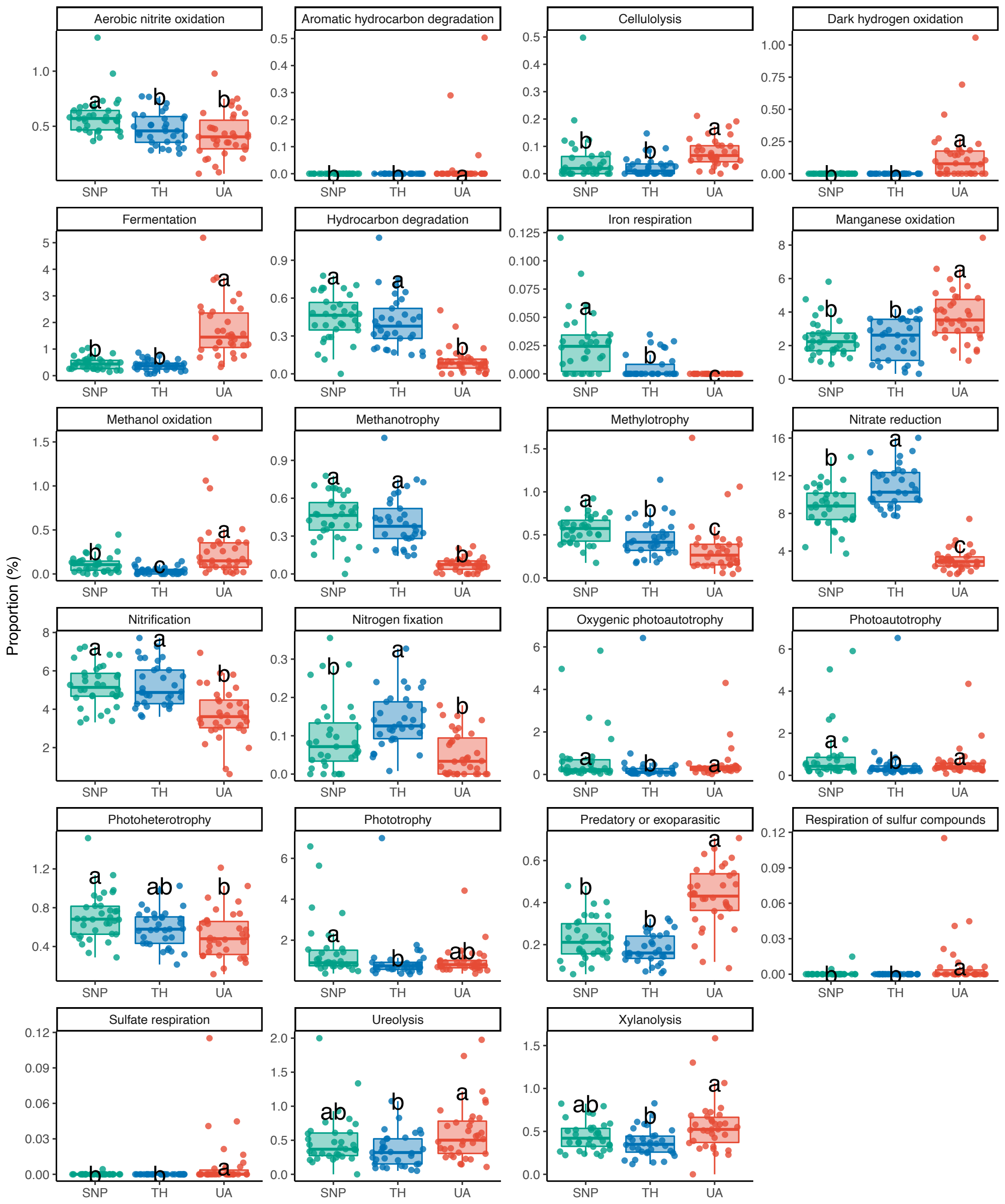


**Figure S2. Changes in the proportions of bacterial/archaeal functional groups across locations.** Different letters indicate significant differences ($P < 0.05$) based on Dunn's test. SNP: Saguaro National Park. TH: Tumamoc Hill. UA: University of Arizona.

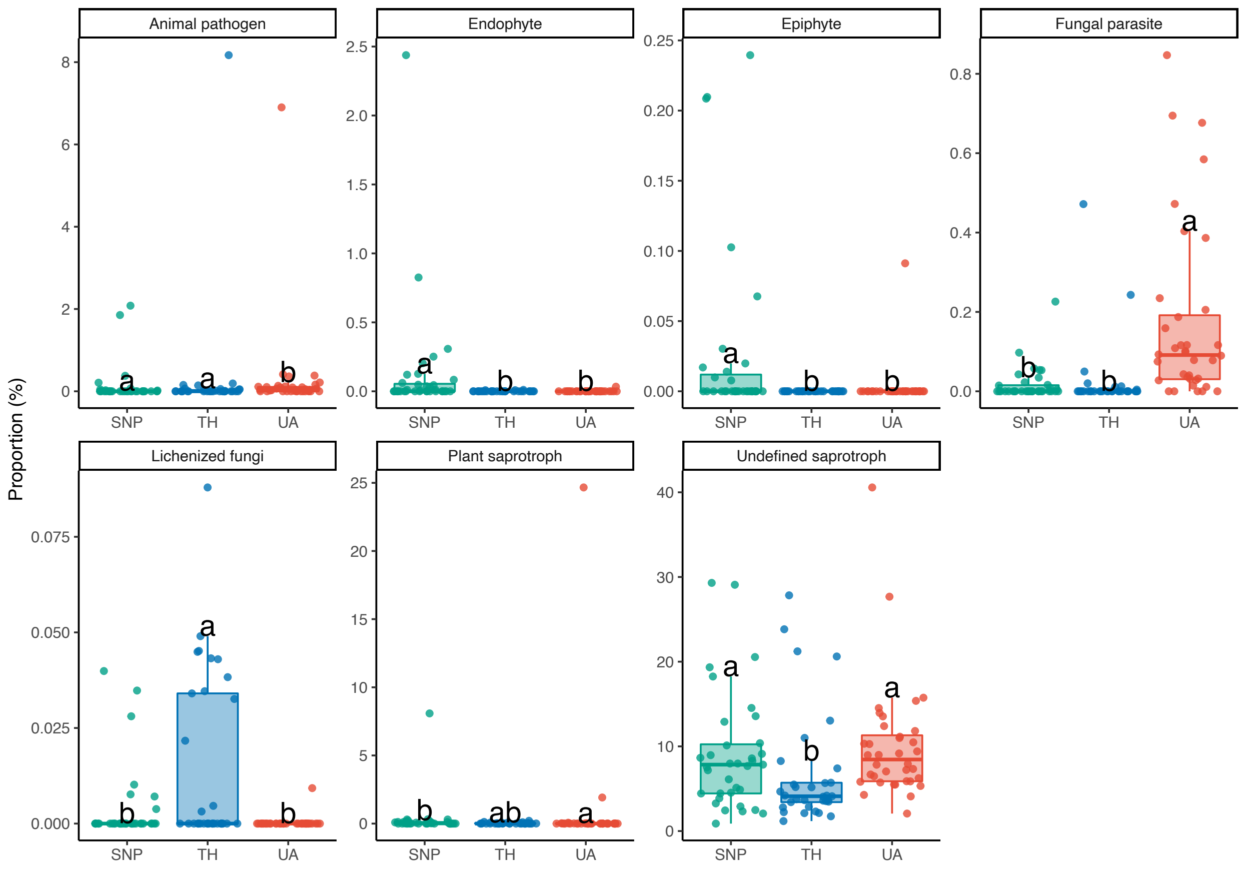


**Figure S3. Changes in the proportions of fungal functional groups across locations.**

Different letters indicate significant differences ($P < 0.05$) based on Dunn's test. SNP: Saguaro National Park. TH: Tumamoc Hill. UA: University of Arizona.
